# FDG PET/CT as a prognostic biomarker in the era of molecular-targeting therapies: max SUVmax predicts survival of patients with advanced renal cell carcinoma

**DOI:** 10.1186/s12885-016-2097-4

**Published:** 2016-02-08

**Authors:** Noboru Nakaigawa, Keiichi Kondo, Ukihide Tateishi, Ryogo Minamimoto, Tomohiro Kaneta, Kazuhiro Namura, Daiki Ueno, Kazuki Kobayashi, Takeshi Kishida, Ichiro Ikeda, Hisashi Hasumi, Kazuhide Makiyama, Yoshinobu Kubota, Tomio Inoue, Masahiro Yao

**Affiliations:** Department of Urology, Graduate School of Medicine, Yokohama City University, 3-9 Fukuura, Kanazawa, Yokohama, 236-0004 Japan; Department of Radiology, Yokohama City University Graduate School of Medicine, Yokohama, Japan; Department of Urology, Yokosuka Kyosai Hospital, Yokosuka, Japan; Department of Urology, Kanagawa Cancer Center, Yokohama, Japan; Department of Urology, Yokohama Minami Kyosai Hospital, Yokohama, Japan

**Keywords:** Renal cell carcinoma, Positron-emission tomography, Computed tomography, Prognosis, Targeted molecular therapy

## Abstract

**Background:**

Various molecular-targeting therapies have become available for the treatment of advanced renal cell carcinoma (RCC). Accurate prognostication is desirable for choosing the appropriate treatment for individual patients. ^18^F-2-fluoro-2-deoxyglucose positron-emission tomography/computed tomography (FDG PET/CT) is a non-invasive tool for evaluating glucose accumulation, which can be an index of biological characteristics of cancer. We prospectively evaluated FDG PET/CT as a prognostic indicator in patients with advanced RCC.

**Methods:**

A total of 101 patients slated for different systematic therapies for advanced RCC were enrolled between 2008 and 2014. A total of 61 patients had recurrent RCC (58 metastatic and 3 regional) and 40 patients had stage IV RCC (36 metastatic and 4 locoregional). Sixteen patients had not undergone nephrectomy. Pre-treatment FDG PET/CT was performed, and the max SUVmax (the highest SUV measurement in each patient) was recorded. The max SUVmax was compared with different clinical risk factors as prognostic indicators. The median observation period was 18 months (range 1–70 months).

**Results:**

The max SUVmax of the 101 subjects ranged from undetectable to 23.0 (median 6.9). Patients with high max SUVmax had a poor prognosis. Multivariate analysis with standard risk factors revealed that max SUVmax was an independent predictor of survival (*p* < 0.001; hazard ratio 1.265; 95 % confidence interval 1.159–1.380). A cutoff of 8.8 for max SUVmax advocated in our previous report was highly significant (*p* < 0.0001). When we subclassified the max SUVmax values, the median overall survival of subjects with max SUVmax < 7.0 was 41.9 months. That of subjects with max SUVmax between 7.0 and 12.0 was 20.6 months. That of subjects with max SUVmax ≥ 12.0 was 4.2 months. The differences were statistically significant.

**Conclusions:**

Pretreatment max SUVmax assessed by FDG PET/CT is a useful prognostic marker for patients with advanced RCC, providing helpful information for clinical decision making.

## Background

Renal cell carcinoma (RCC) accounts for 3 % of all adult cancers [1]. Approximately 30 % of RCC patients are diagnosed with metastases, and an additional 20–40 % develop metastases after radical nephrectomy with curative intent [2, 3]. Cytokine therapies have been the only treatments available for advanced RCC for a long time, and have been associated with a disappointing outcome [4, 5]. With elucidation of the oncogenic mechanisms of RCC, however, agents that target critical molecules in the biological pathways necessary for oncogenesis, such as vascular endothelial cell growth factor or the mammalian target of rapamycin (mTOR), have been developed. These molecular-targeting therapeutics have improved outcomes for patients with advanced RCC [6–9], and are recommended as the main treatments for advanced RCC in clinical guidelines applied worldwide [10, 11].

It is well known that prognoses for patients with RCC can vary, and the guidelines recommend risk-directed therapies using prognostic classifications based on a combination of clinical information and laboratory data [8, 10, 11]. The Memorial Sloan-Kettering Cancer Center (MSKCC) classification using five clinical factors including performance status, the interval from diagnosis to start of treatment, lactate dehydrogenase (LDH), corrected calcium, and anemia, is most commonly used for prognosis [12]. These clinical parameters are thought to express the biological activity of RCC indirectly. However, in this era of molecular-targeting therapy, an index that expresses the biological activity of RCC directly, and prognosticates accurately, is desired for appropriate clinical decision making.

^18^F-2-fluoro-2-deoxyglucose positron emission tomography-computed tomography (FDG PET/CT) is a useful non-invasive tool for evaluating glucose metabolic status, which can be an index of the biological activity of cancer. We focused on standardized uptake value (SUV), a quantitative simplified measure of tissue FDG accumulation, and previously reported that max SUVmax (i.e., the highest SUV of all RCC lesions in each patient) predicted the overall survival (OS) of patients with advanced RCC [13]. In that paper, we reported that the survival of patients with max SUVmax greater than or less than the cutoff value of 8.8 were statistically different (*p*=0.0012). Subsequently, Kayani reported that high SUVmax correlated with shorter overall survival in patients treated with the tyrosine kinase inhibitor (TKI) sunitinib [14]. Chen reported that baseline SUVmax correlated with the overall survival of patients with RCC treated by everolimus, which is an oral mTOR inhibitor (mTORI) [15]. Other investigators also advocated the usefulness of FDG PET/CT as a prognostic tool for patients with RCC [16, 17].

In this study, we report results from an expanded number of patients and a longer follow-up period.

## Methods

### Patients

This was a prospective study that followed enrolled patients slated to undergo systemic therapies for pathologically proven advanced RCC between June 2008 and January 2014. The patients were initially assessed by conventional imaging techniques (computed tomography, magnetic resonance imaging, or bone scintigraphy) and diagnosed as stage IV or recurrent RCC. Patients with uncontrolled diabetes mellitus (fasting blood sugar > 150 mg/dL), other known malignancies, and patients who had received treatment within 2 weeks prior to enrollment were excluded. The study protocol was approved by the Yokohama City University Institutional Review Board. Written informed consent was obtained from all patients.

Initially, 110 patients were enrolled in the study. Nine were eventually eliminated: four whose pathology could not be confirmed conclusively, three who decided against treatment after evaluation by FDG PET/CT, one patient had a fasting blood sugar over 150 mg/dL, and one with contralateral kidney metastases for which accurate SUV could not be measured owing to the urinary excretion of the radiotracer. This left a total of 101 patients for the analysis, including 24 who had been analyzed in the preliminary report [12]. The first therapeutic interventions after enrollment in this study were decided before the evaluation by FDG PET/CT.

### Imaging

Patients fasted for at least 6 h prior to intravenous injection of ^18^F FDG. PET/CT images were acquired (Aquiduo 16^®^; Toshiba Medical Systems, Tokyo, Japan). One hour after injection of 2.5 MBq/kg of ^18^F FDG, PET/CT images were acquired from the top of the head to the mid-thigh. A low-dose, non-contrast CT scan was acquired first and used for attenuation correction. Emission images were acquired in three-dimensional mode for 2 min per bed position. After PET acquisition, contrast-enhanced CT was performed with a 2-mm section thickness, 120 kV, 400 mA, 0.5 s/tube rotation, from the top of the head to the mid-thigh, with breath holding. A total of 100 mL contrast medium (iopamidol) was administered intravenously at a rate of 1.0 mL/s. The scan delay was set at 120 s after the start of the injection of contrast material. Patients with serum creatinine levels > 1.5 mg/dL were examined without contrast material. The all cases with origin RCC were evaluated by contrast enhanced CT scan. Images were reconstructed by attenuation-weighted, ordered-subset expectation maximization (four iterations, 14 subsets, 128 × 128 matrix, with 5-mm Gaussian smoothing). The SUV was determined according to the standard formula, with activity in the volume of interest (VOI) recorded as Bq per mL/injected dose in Bq per total body weight (kg). VOIs were positioned to encompass targets within areas of increased uptake and measured on each slice by two experienced physicians (DU and KM), who were blinded to clinical data. Discrepancies were resolved by consensus reading. Analysis of FDG uptake in the primary tumor was made with reference to contrast-enhanced CT images to differentiate tumor from physiologic parenchymal and urinary tract activity. The maximum activity of all VOIs of each patient was defined as the max SUVmax.

### Statistical analysis

Survival time was calculated from the date of evaluation by ^18^F-FDG PET/CT to the date of death. A Cox proportional hazards model was used to assess the effects of max SUVmax on survival. OS curves were estimated by the Kaplan-Meier method, and the resulting curves were compared using the log-rank test. The impacts on overall survival (OS) of max SUVmax and other standard clinicopathologic factors (performance status, the interval from diagnosis to start of treatment, LDH, corrected calcium, age, sex, and pathology) were analyzed by a univariate Cox hazard model, and the factors with *p* < 0.05 were analyzed by a multivariate Cox hazard model. All statistical analyses were carried out with commercial software (SPSS^®^, SPSS Inc., Chicago, IL, USA).

## Results

### Patient characteristics

The characteristics of the 101 patients are shown in Table 1. Of 40 patients with Stage IV disease, 24 had not undergone prior nephrectomy. The FDG PET/CT evaluation of the 17 patients who had received prior therapy was performed more than 2 weeks after the end of any previous treatment.

**Table 1:** Patients characteristics

Characteristic	No. of patients (%)		
No. of patients	101		
Sex			
Male	83		(82)
Female	18		(18)
Age, years			
Median (Range)	65	(32–82)	
Pathology			
Clear cell	86		(85)
Papillary	6		(6)
Clear cell/Sarcomatoid	4		(4)
Sarcomatoid	2		(2)
Hemodialyssis	2		(2)
Unclassified	1		(1)
Prior nephrectomy			
Yes	77		(76)
No	24		(24)
Disease status			
Recurrent	61		(60)
Metastatic		58	(57)
Regional		3	(3)
Stage IV	40		(40)
Locoregional		4	(4)
Metastatic		36	(36)
Prior systematic Therapy			
Yes	17		(17)
IFN-α		9	
IFN-α/sorafenib		2	
Sorafenib		2	
Sunitinib		1	
S-1		1	
IFN-α/UFT		1	
Sorafenib/Temsirolimus		1	
No	84		83

*Abbreviation: IFN-α* interferon-α

### Interventions

After evaluation by PET/CT, 40 patients were treated with sorafenib, 38 with sunitinib, 12 with interferon-α (IFN-α), eight with temsirolimus, one with axitinib, one with pazopanib, and one with chemotherapy. Comprehensive decisions regarding first interventions were made from pathological and clinical information before the FDG PET/CT evaluation.

The median observation period was 18 months (range 1–70). During the observation period, 44 patients were treated with a single intervention (20 sorafenib, 19 sunitinib, four IFN-α, and one temsirolimus), 22 with two interventions (10 TKI to mTORI, 9 TKI to TKI, and 3 mTORI to TKI), 20 with three interventions, and 15 with more than three interventions. Ninety-six patients were treated with TKI, 43 with mTORI, and 16 with IFN-α. Six patients underwent metastasectomy, and five, nephrectomy (Table 2). There were 57 cases of death due to cancer; we confirmed that the other 44 patients were still alive at the time of this writing. There were no cases of death due to other causes.

**Table 2:** Interventions after PET/CT evaluation

Interventions	No. of patients		(%)
Single intervention	44		(44)
Sunitinib		19	
Sorafenib		20	
IFN-α		4	
Temsirolimus		1	
2 interventions	22		(22)
TKI to mTORI		10	
TKI to TKI		9	
mTORI to TKI		3	
3 interventions	20		(20)
3 < interventions	15		(15)
TKI	96		(95)
mTORI	39		(39)
IFN-α	16		(16)
Metastasectomy	6		(6)
Nephrectomy	5		(5)

*Abbrebiations: IFN-α* interferon-α, *TKI* tyrosine kinase inhibitor, *mTORI* mTOR inhibitor

### Assessment by FDG PET/CT

**Fig. 1:**
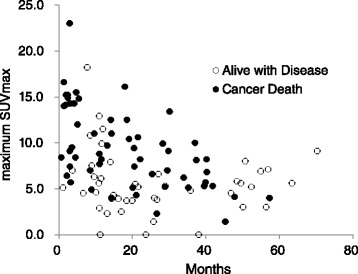
The association of pretreatment max SUVmax and survival. The vertical axis plots the pretreatment max SUVmax of individual patients, and the horizontal axis plots their survival. Open circles are the patients who were alive on the last observation days and closed circles are the patients dead as a result of cancer

The max SUVmax of all patients ranged from undetectable to 23.0 (median 6.9). When max SUVmax was analyzed as a continuous variable, high max SUVmax was associated with shorter OS, (Fig. 1) (*p* < 0.001, hazard ratio 1.257, 95 % confidence interval [CI] 1.177–1.342).

The impact of max SUVmax on OS was compared with that of numerous standard risk factors. The multivariate analysis of max SUVmax with performance status, LDH, corrected calcium, interval between diagnosis and entry, and pathology (*p* < 0.05 in univariate analysis) revealed that max SUVmax was a significant independent predictor of survival (Table 3).

**Table 3:** Univariate and multivariate Cox analyses of max SUVmax versus standard prognostic factors for advanced RCC

	Univariate cox analyses			Multivariate cox analyses		
Risk Fcotr	P value	HR	95 % CI	P value	HR	95 % CI
max SUVmax (continuous variable)	<0.001	1.257	1.177-1.342	<0.001	1.265	1.159-1.380
Karnofsky performance status (<80 %)	0.036	2.107	1.051–4.221	0.296	0.623	0.256–1.514
Lactate dehydrogenase (>1.5x upper limit of normal)	<0.001	8.655	3.559–21.049	0.001	5.026	1.935–13.052
Corrected calcium (>10 mg/dl)	0.014	2.457	1.198–5.038	0.151	1.943	0.784–4.815
Hemoglobin (<lower limit of normal)	0.121	1.810	0.854–3.833			
Interval from initial diagnosis to treatment (<1 year)	0.014	1.937	1.142–3.286	0.164	1.549	0.836–2.870
Age (>65 years old)	0.416	0.803	0.474–1.361			
Sex (male or female)	0.890	1.046	0.551–1.984			
Pathology (clear or non–clear)	0.044	2.113	1.021–4.373	0.962	0.980	0.419–2.291

**Fig. 2:**
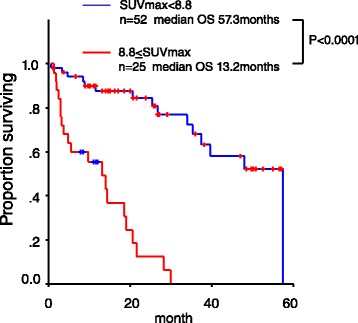
Validation of the cutoff point used in the preliminary report. We validated the usefulness of max SUVmax 8.8 [12], focusing on the 77 patients who were enrolled after the preliminary analysis

At first, we validated the application of a cutoff of max SUVmax of 8.8, which was the same cutoff point for OS prediction used in our previous report [13], focusing on the 77 patients who were enrolled after the preliminary analysis. The median OS of the 52 patients with RCC having a max SUVmax < 8.8 was 57.3 months, and that of the 25 patients with RCC having the max SUVmax ≥ 8.8 was 13.2 months (95 % CI 5.89–20.51) (*p* < 0.0001) (Fig. 2).

**Fig. 3:**
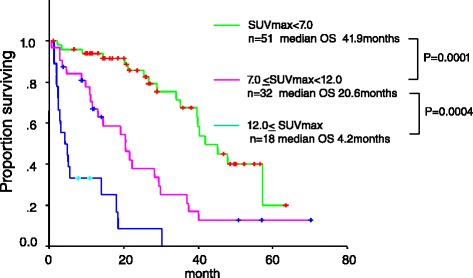
Overall survival curve of total 101 patients stratified by two cutoff points, max SUVmax 7.0 and 12.0

We then divided the 101 patients into three subgroups by max SUVmax. Because the existence of the subgroup of patients with RCC showing very high max SUVmax whose survival time were less than 1 year became apparent in Fig. 1. The max SUVmax of 51 patients (50 %) was < 7.0 and the median OS of this subgroup was 41.9 months (95 % CI 34.12–49.68). The max SUVmax of 32 patients (32 %) were ≥ 7.0 and < 12.0, and median OS was 20.6 months (95 % CI 12.4–28.8). The max SUVmax of 18 patients (18 %) was ≥ 12.0, and median OS was 4.2 months (95 % CI 0.7–7.7). Differences in OS for these patient subgroups were statistically significant (< 7.0 vs. ≥ 7.0 and < 12.0: *p*=0.0001, ≥ 7.0 and < 12.0 vs. ≥ 12.0: *p*=0.0004) (Fig. 3).

**Fig. 4:**
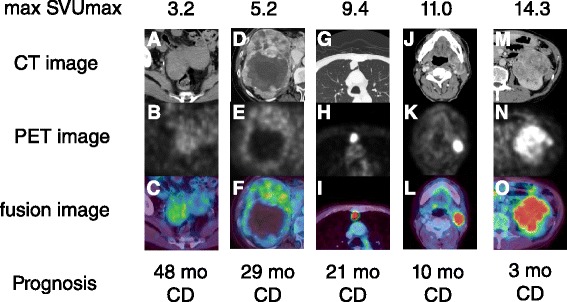
The features of FDG PET/CT and prognosis. (*a*, *b*, *c*) A case with ovarian recurrence and a max SUVmax of 3.2. (*d*, *e*, *f*) A case with a primary tumor and a max SUVmax of 5.2. (*g*, *h*, *i*) A case with lung metastasis and a max SUVmax of 9.4. (*j*, *k*, *l*) A case with submandibular lymph node metastasis and a max SUVmax of 11.0. (*m*, *n*, *o*) A case with a primary tumor and a max SUVmax of 14.3. *a d g j m*: CT imaging. *b e h k n*: PET images. *c f i l o*: fusion images. mo=month. CD=cancer death

Figure 4 presents the features of the FDG PET/CT scans. Regardless of the tumor size and the organs where the metastasis was located, the patients with lower max SUVmax exhibited better OS than the patients with higher max SUVmax.

## Discussion

We demonstrated that max SUVmax by FDG PET/CT is a useful prognostic marker for survival of patients with advanced RCC. It is reasonable that RCCs with high max SUVmax would have poorer prognoses because it has been suggested that RCCs with rapid progression need more glucose as an energy source and take up more FDG. Numerous recent studies of various types of cancer, including head-and-neck, lung, and cervical, have explored the prognostic significance of the SUV [18–21]. Although the size of our study was relatively small, the results were more significant compared with these studies of other malignancies. We propose two possible reasons for this result. The first is that the prognosis of patients with advanced RCC can vary widely. Many researchers have been trying to establish methods to predict the prognosis of RCC. The MSKCC classification advocated by Motzer et al. is most commonly used for prognosis [12], and they reported median OS of favorable, intermediate, and poor risk patients of 30, 14, and 5 months, respectively, when the patients were divided into the three groups by five clinical risk factors. The second reason why results may have been more significant in our study was that the main treatments were targeted molecular therapeutics, which suppressed the biological activity of the cancer and the original biological properties of RCC affected the clinical courses markedly. We showed that the power of prediction by max SUVmax was superior to that by the risk factors used for MSKCC classification. It is meaningless, however, to discuss whether evaluation by FDG PET/CT or by clinical factors is better. We must focus on tailoring treatment according to prognosis to lengthen OS.

FDG PET/CT has not been generally applied to evaluate RCC owing to the urinary excretion of the radiotracer, which can mask the presence of primary lesions [22, 23]. However we previously reported that FDG accumulation was evaluable in 94.9 % of all RCC lesions diagnosed by a CT scan except for lung or liver metastases < 1 cm, providing combined morphological and functional information [13]. These results were consistent with a previous report [24]. Additionally, Majhail et al. proved the pathological accuracy of diagnosis by FDG PET [25]. They performed biopsy or surgical resection of 36 distant metastatic lesions in 24 patients and revealed that the pathological positive predictive value of FDG PET/CT was 100 %.

Recently, we and other researchers have reported using FDG PET/CT to assess the response of RCC to molecular-targeting therapies [14, 26–28]. This evaluation is clinically beneficial because molecular-targeting therapies, as opposed to classical cytotoxic anticancer therapeutics, do not always cause obvious tumor shrinkage. These studies have reported that the decrease of FDG uptake predicted long-term dormancy of RCC, suggesting that FDG uptake can be used not just as a prognostic indicator before treatment, but also to assess the real-time status of biological activity in RCC. When more data about the assessment of RCC by FDG PET/CT are accumulated in the future, the therapeutic strategy of the individual patient with advanced RCC may be decided based on the assessment by FDG PET/CT. For example, the patients with RCC showing very high max SUVmax will be treated with temsirolimus,, which is an intravenous mTOR inhibitor currently recommended for patients who are classified as “poor risk” by clinical risk factors. In contrast, patients with RCC showing low max SUVmax may be treated with more flexibility, focusing on the quality of life with the careful sequential assessment by FDG PET/CT.

Our study has several limitations. First, the patients were treated with various therapies, including TKI, mTORI, and IFN-α after evaluation by FDG PET/CT. Second, the number of systematic treatments that the patients enrolled in this study were subjected to were variable. Third, five patients underwent nephrectomies and six patients underwent metastasectomy, although the purpose of these surgeries was not complete resection.

The pretreatment max SUVmax assessed by FDG PET/CT can predict survival of patients with advanced RCC. FDG PET/CT has the potential to provide helpful information for clinical decision making. Future pathological and molecular studies are needed to disclose the biological means of FDG accumulation in RCC.

## Conclusions

Pretreatment max SUVmax assessed by FDG PET/CT can predict survival of patients with advanced RCC. FDG PET/CT has the potential to be an “imaging biomarker,” providing helpful information for clinical decision making.

## Abbreviations

CI: confidence interval
FDG PET/CT: ^18^F-2-fluoro-2-deoxyglucose positron-emission tomography/computed tomography
IFN-α: interferon-α
LDH: lactate dehydrogenase
MSKCC: Memorial Sloan-Kettering Cancer Center
mTOR: mammalian target of rapamycin
mTORI: inhibitor of mammalian target of rapamycin
OS: overall survival
RCC: renal cell carcinoma
SUV: standardized uptake value
TKI: tyrosine kinase inhibitor
VOI: volume of interest
